# Miscellany

**Published:** 1897-09

**Authors:** 


### Miscellany.

### STUDY OF THE AMERICAN MEDICINAL FLORA.

The sub-commission of the Pan-American Medical Congress appointed to study the medicinal plants of the United States has entered into an association with the Smithsonian Institution for that purpose. The attention of our readers is called to the respective circulars issued by these organisations, which we print below :

Smithsonian Institution,
Washington, D. C., May 28, 1897.

*Dear Sir*—The Smithsonian Institution has undertaken to bring together all possible material bearing on the medicinal uses of plants in the United States. Arrangements have been made with a body representing the Pan-American Medical Congress, the sub-commission on medicinal flora of the United States, to elaborate a report on this subject, and the material when received will be turned over to them for investigation.

The accompanying detailed instructions relative to specimens and notes have been prepared by the sub-commission.

All packages and correspondence should be addressed to the Smithsonian Institution, Washington, D. C., and marked on the outside medicinal plants for the United States National Museum. Franks, which will carry specimens, when of suitable size, together with descriptions and notes, free of postage through the mails, will be forwarded upon application. Should an object be too large for transmission by mail, the sender is requested, before shipping it, to notify the institution, in order that a proper authorisation for its shipment may be made out. Respectfully,

(Signed) S. P. LANGLEY, *Secretary.*

INSTRUCTIONS RELATIVE TO MEDICINAL PLANTS.

The Pan-American Medical Congress, at its meeting held in the City of Mexico in November, 1896, took steps to institute a systematic study of the American medicinal flora, through the medium of a general commission and of special sub-commissions, the latter to be organised in the several countries. The sub-commission for the United States has been formed and consists of Dr. Valery Havard, U. S. A., chairman; Mr. Frederick V. Coville, botanist of the United States department of agriculture; Dr. C. F. Millspaugh, curator of the botanical department of the Field Columbian Museum, Chicago; Dr. Charles Mohr, state botanist of Alabama; Dr. W. P. Wilson, director of the Philadelphia commercial museums; and Prof. H. H. Rusby, of the New York College of Pharmacy. This sub-commission solicits information concerning the medicinal plants of the United States from every one in a position to accord it. The principal points of study are as follows:

(1) Local names; (2) local uses, together with historical facts; (3) geographical distribution and degree of abundance in the wild state; (4) is the plant collected for market and, if so, (*a*) at what season of the year? (*b*) to how great an extent? (*c*) how prepared for market? (*d*) what is the effect of such collection upon the wild supply? (*e*) what price does it bring? (*f*) is the industry profitable? (5) is the plant, or has it ever been, cultivated, and if so, give all information on the subject, particularly as to whether such supplies are of superior quality and whether the industry has proved profitable; (6) if not cultivated, present facts concerning the life history of the plant which might aid in determining methods of cultivation; (7) is the drug subjected to substitution or adulteration, and if so, give information as to the plants used for this purpose.

While it is not expected that many persons will be able to contribute information on all these points concerning any plant, it is hoped that a large number of persons will be willing to communicate such partial knowledge as they possess.

It is not the important or standard drugs alone concerning which information is sought. The sub-commission desires to compile a complete list of the plants which have been used medicinally, however trivial such use may be. It also desires to collect all obtainable information, historical, scientific and economic, concerning our native and naturalised plants of this class, and, to that end, invites the coöperation of all persons interested. Poisonous plants of all kinds come within the scope of our inquiry, whether producing dangerous symptoms in man or simply skin inflammation, or, as "loco-weeds," deleterious to horses, cattle and sheep. In this respect the general reputation of a plant is not so much desired as the particulars of cases of poisoning actually seen or heard from reliable observers. It is believed that much interesting knowledge can be obtained from Indians, Mexicans and half-breeds, and that, consequently, Indian agencies and reservations are particularly favorable fields for our investigation. Such knowledge will be most acceptable when based upon known facts or experiments.

In order to assist in the study of the habits, properties and uses of medicinal plants, the sub-commission undertakes to furnish the name of any plant specimen received, together with any desired information available.

Owing to the diversity in the common names of many plants it will be necessary for reports, when not furnished by botanists or others qualified to state the botanical names with certainty, to accompany the same with some specimen of the plant sufficient for its identification. While the sub-commission will endeavor to determine the plant from any portion of it which may be sent, it should be appreciated that the labor of identification is very greatly decreased, and its usefulness increased, by the possession of complete material, that is, leaf, flower and fruit, and in the case of small plants, the underground portion also. It is best to dry such specimens thoroughly in a flat condition, under pressure, before mailing. While any convenient means for accomplishing this result may be employed, the following procedure is recommended: Select a flowering or fruiting branch, as the case may be, which when pressed shall not exceed 16 inches in length by 10 inches in width. If the plant be a herb 2 or 3 feet high, it may be doubled to bring it within these measurements. If it possess root leaves, some of these should be included. Lay the specimen flat in a fold of newspaper and place this in a pile of newspapers, carpet felting, or some other form of paper which readily absorbs moisture and place the pile in a dry place under a pressure of about 20 to 30 pounds, sufficient to keep the leaves from wrinkling as they dry. If a number of specimens are pressed at the same time, each is to be separated from the others by 3 or 4 folded newspapers or an equivalent in other kinds of paper. In 12 to 24 hours these papers will be found saturated with the absorbed moisture and the fold containing the specimen should be transferred to dry ones. This change should be repeated for from 2 to 5 days according to the state of the weather, the place where the drying is done, the fleshiness of the specimens, and the like. The best way to secure the required pressure is by means of a pair of strong straps, though weights will do. The best place for drying is beside a hot kitchen range. When dry the specimens should be mailed between cardboards or some other light but stiff materials which will not bend in transit.

It is a most important matter that the name and address of the sender should be attached to the package and that the specimens, if more than one, should be numbered, the sender retaining also specimens bearing the same number, to facilitate any correspondence which may follow. The sub-commission requests that, so far as practicable, all plants sent be represented by at least four specimens.

(Signed) H. H. RUSBY, M. D.,
*Chairman of the General Commission, New York College of Pharmacy.*

VALERY HAVARD, M. D.,
*Chairman of the Sub-Commission, Fort Slocum, Davids Island, N. Y.*

---

A LARGE proportion of what are known as second-hand corks are said on good authority to contain disease germs (*New York Journal*). Most of the second-hand corks are gathered in various parts of the city by scavengers. It makes no difference to the scavenger how vile the spot may be where the cork is found. It may come out of a cesspool or sewer, from a garbage heap, an ash barrel or bar-room sweepings. It's all the same to the scavenger. All he seeks is the filling of his pail with corks, whether they be black with dirt or of the cleaner variety. For a pailful he receives from twenty-five to thirty cents. The purchaser is some East side cork dealer, who washes them over with muriatic acid and brightens their appearance. Then he in turn sells the corks to second-class bottlers for from ten to twelve cents a gross.

These bottlers may be found in great number in the thickly populated districts of the city. Many of their shops are located in basements or cellars. Some of these basements and cellars are damp and unhealthy. Then certain bottlers have their stables on the same floor, in the same room, where the bottling is done. Soda water bottled in these places is sold for a cent a glass or bottle. Its varied coloring is due to the use of aniline. The manufacturer uses on an average five pounds of sugar to make a gallon of syrup. Often glucose is used instead, although saccharine is coming into favor as a cheap substitute for sugar. About an ounce of syrup is used to each bottle of soda water.

The second-hand corks are soaked in water, so that they can be driven into the bottles. This forcing process sends all the filth and dirt, still on the cork, into the soda water in the bottle, and this mixture is what thousands of men, women and children of the tenement districts are drinking under the name of soda water, sarsaparilla and ginger ale.

